# Case Report: The University of Michigan Dioxin Exposure Study: A Follow-up Investigation of a Case with High Serum Concentration of 2,3,4,7,8-Pentachlorodibenzofuran

**DOI:** 10.1289/ehp.0901723

**Published:** 2010-04-23

**Authors:** Alfred Franzblau, Elizabeth Hedgeman, Olivier Jolliet, Kristine Knutson, Tim Towey, Qixuan Chen, Biling Hong, Peter Adriaens, Avery Demond, David H. Garabrant, Brenda W. Gillespie, James Lepkowski

**Affiliations:** 1 Department of Environmental Health Sciences, University of Michigan School of Public Health, Ann Arbor, Michigan, USA; 2 LimnoTech, Ann Arbor, Michigan, USA; 3 Department of Biostatistics, School of Public Health; 4 Department of Civil and Environmental Engineering, College of Engineering and; 5 Institute for Social Research, University of Michigan, Ann Arbor, Michigan, USA

**Keywords:** dioxins, food, furans, pathway of exposure, polychlorinated biphenyls

## Abstract

**Context:**

Polychlorinated dibenzo-*p*-dioxins, polychlorinated dibenzofurans, and dioxin-like polychlorinated biphenyls that have toxic equivalency factors (TEFs) were measured in serum of 946 subjects in five Michigan counties. The study was motivated by concerns about human exposure to dioxin-contaminated sediments in the Tittabawassee River (TR). Most of the toxic equivalency in TR sediments is from two furan congeners, 2,3,7,8-tetrachlorodibenzofuran and 2,3,4,7,8-pentachlorodibenzofuran (2,3,4,7,8-pentaCDF).

**Case presentation:**

The individual with the highest adjusted (for age, age squared, and body mass index) serum level of 2,3,4,7,8-pentaCDF in the study (42.5 ppt) reported a unique history of raising cattle and vegetables in the floodplain of the TR. Interviews and serum samples were obtained from the index case and 15 other people who ate beef and vegetables raised by the index case. 2,3,4,7,8-pentaCDF in beef lipid was estimated to have been more than three orders of magnitude greater than background (1,780 vs. 1.1 ppt). The mean, median, and 95th percentile for serum 2,3,4,7,8-pentaCDF in the study control population were 6.0, 5.4, and 13.0 ppt, respectively, and were 9.9, 8.4, and 20.5 ppt among beef and vegetable consumers, respectively. Back extrapolation for the index case suggests that his increase in serum concentration of 2,3,4,7,8-pentaCDF above background may have been as high as 146 ppt.

**Discussion:**

Consumption of beef and/or vegetables raised on dioxin-contaminated soil may be an important completed pathway of exposure.

**Relevance to public health practice:**

Animals and crops should not be raised for human consumption in areas contaminated with dioxins.

Polychlorinated dibenzodioxins (PCDDs) and polychlorinated dibenzofurans (PCDFs) are unintended by-products of certain chemical processes involving chlorine and incineration processes [Agency for Toxic Substances and Disease Registry (ATSDR) 1994, 1998]. Some major examples include the bleaching processes involved in making white paper products, manufacturing chlorinated phenols, incinerating waste, producing various metals, and the burning of fossil fuels (ATSDR 1994, 1998). PCDDs of natural origin have also been discovered in certain clays and can lead to human exposure (Ferrario and Byrne 2000; Franzblau et al. 2008). Production or combustion of polychlorinated biphenyls (PCBs) is another source of PCDFs (ATSDR 1994). Commercial production of PCBs ended in the United States in 1977 (ATSDR 2000). Collectively referred to as dioxins, or dioxin-like compounds, PCDDs, PCDFs, and PCBs were spread widely in the environment during the last century, largely as a result of human activities.

The dominant source of exposure to dioxin-like compounds in the general population is food (> 90%), mostly by consuming animal products (ATSDR 1994, 1998, 2000). Farmers and other persons who consume foods raised in contaminated areas are at risk of exposure (Ewers et al. 1996, 1997).

As part of the University of Michigan Dioxin Exposure Study (UMDES) the 29 congeners of PCDDs, PCDFs, and dioxin-like PCBs that have consensus toxic equivalency factors (TEFs) were measured in serum of 946 subjects who were a representative sample of the general population in five Michigan counties, including 251 participants from two control counties located > 100 miles away from the Dow facilities. The study was motivated by concerns about possible human exposure to dioxin-contaminated sediments in the Tittabawassee River (TR) believed to be the result of historical industrial activities of the Dow Chemical Company (Figure 1). Most (~ 80%) of the total toxic equivalency (TEQ) in TR floodplain (FP) sediments is due to two furan congeners, 2,3,7,8-tetrachlorodibenzofuran (2,3,7,8-TCDF) and 2,3,4,7,8-pentachlorodibenzofuran (2,3,4,7,8-pentaCDF) (Demond et al. 2008; Hilscherova et al. 2003). The half-life of 2,3,7,8-TCDF is short; thus, it is less likely to accumulate in humans; 2,3,4,7,8-pentaCDF, on the other hand, has a prolonged serum half-life in humans (~ 7 years) (Milbrath et al. 2009). Because it can accumulate in human tissues, 2,3,4,7,8-pentaCDF can serve as a bio-marker of remote exposure to contaminated TR sediment. One individual with the highest adjusted serum level of 2,3,4,7,8-pentaCDF in the UMDES [42.5 ppt lipid adjusted, or 4.29 studentized residuals above the log-normalized mean of the control population after adjusting for age, age squared, and body mass index (BMI)] reported a unique exposure history that involved consuming homegrown beef and vegetables raised in the TR FP.

In this report, we have described this person’s serum results and the results of a followup investigation that involved friends and family members who also reported regularly eating the beef and vegetables that were raised on this individual’s property.

The UMDES used a two-stage clustered random sampling design to recruit subjects from five counties in the State of Michigan (USA). Eligible participants were required to be at least 18 years old and to have lived in their homes for at least 5 years. For the main study, eligible subjects were interviewed for 1 hr, and blood, house dust, and soil samples were collected for chemical analyses. The collection of field data for the subjects was completed between 2004 and 2005, and the samples were analyzed for PCDDs, PCDFs, and PCBs by Vista Analytical Laboratory (El Dorado Hills, CA, USA) using modified U.S. Environmental Protection Agency (EPA) Methods 8290 and 1668 (U.S. EPA 1994 1999). Serum results are reported in parts per trillion on a lipid-adjusted basis, and soil results are reported in parts per trillion on a dry weight basis. Serum total lipids for each sample were calculated using the Phillips formula summing triglycerides and total cholesterol. TEQ values were calculated using 2005 TEFs (Van den Berg et al. 2006). More complete methodological details of the parent study are available elsewhere (Demond et al. 2008; Garabrant et al. 2009a, 2009b).

Here, we refer to the person with the highest adjusted serum level of 2,3,4,7,8-pentaCDF in the UMDES as the index case. As part of an effort to better understand why the serum level of 2,3,4,7,8-pentaCDF for the index case was elevated, we conducted a follow-up interview to better understand his unique dietary history of consuming homegrown beef and vegetables from the TR FP. The index case identified friends and family members who were most likely to have regularly consumed beef and vegetables raised on his property (*n* = 15). These subjects were interviewed about diet (particularly consumption of beef and vegetables from the TR FP), occupation, residential history, personal habits (e.g., smoking), height, weight and change in weight, breast-feeding, hobbies, and recreational activities in or near the TR. Subjects were also invited to undergo phlebotomy to measure PCDDs, PCDFs, and dioxin-like PCBs in serum, except the index case and case 5 who had already undergone blood testing as part of the UMDES and were not retested. All subjects provided written consent that had been approved by the University of Michigan Health institutional review board.

Because the index case stopped raising beef in 1996 and the follow-up investigation was performed in 2008, no beef samples were available for analysis. The 2,3,4,7,8-pentaCDF beef concentration was therefore estimated on the basis of local environmental concentrations in vegetation and soil and experimental biotransfer factors from the literature. The cattle were raised for an average of 18 months, but because of variation in dietary sources of fodder (see below), we assumed (conservatively) that cattle consumed fodder from the FP for only 6 months. The daily intake of 2,3,4,7,8-pentaCDF by the cattle in the FP (*I*) was estimated as


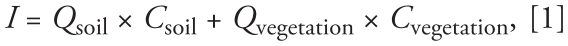


where *C*_soil_ and *C*_vegetation_ (in picograms per gram dry weight) are the observed soil and vegetation concentrations in the FP, and *Q*_soil_ and *Q*_vegetation_ (grams dry weight per day) are the daily quantities of local soil and fodder ingested by the cattle. Because the cattle roamed widely on the property of the index case, and only one sample of soil and vegetation was taken from each property in the FP, the concentration of 2,3,4,7,8-pentaCDF in soil and vegetation eaten by the cattle was estimated by averaging FP results from six properties (including the index case and the five closest properties in the study that front on the same side of the river within ~ 1 km of the index case’s property). Daily intake of fodder by cattle was estimated as *Q*_vegetation_ = 7,200 g dry weight/day, with an expected range of 5,000–10,000 g dry weight/day [International Atomic Energy Agency (IAEA) 1994]. Soil ingestion is expressed as 6% of feed or forage intake for grazing cattle, yielding *Q*_soil_ = 432 g dry weight/day (IAEA 1994). Feil et al. (2000) reported that intake by cattle of a daily dose of 83.3 ng/day for 4 months resulted in an average increase in lipid-adjusted concentration of 2,3,4,7,8-pentaCDF of 105 pg/g lipid in beef at the end of the experiment (based on Feil et al. 2000, their Figure 2 and Tables 4 and 5). Because the cattle raised in the FP had been grazing there for at least 6 months, the estimates from Feil et al. (2000) can be used to calculate a conservative estimate of the resulting increase in the 2,3,4,7,8-pentaCDF concentration in beef (Δ*C*) for a given intake level (*I*):


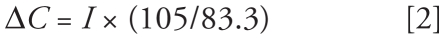


Because the human serum concentrations in this study were measured ≥ 9 years after beef consumption had ended, we estimated the decrease in serum concentration that may have occurred after consumption stopped. An attenuation factor can be calculated using the following equation:


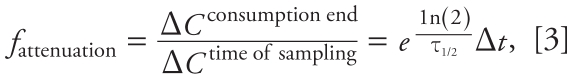


where Δ*t* (year) is the elapsed time between the end of the beef ingestion and measurement of serum concentration, Δ*C* is the increase in serum concentration of 2,3,4,7,8-pentaCDF at either the time consumption of contaminated food ended (numerator) or the time that serum was sampled (denominator), and τ_1/2_ (years) is the average elimination half-life during this period, calculated according to Milbrath et al. (2009).

## Case Presentation

The index case owned property in the TR FP that fronted on the river. His recollection, as corroborated by the friends and family members, was that in about 1984 or 1985 he began to raise cows on his land. Each year in the spring, he bought two or three calves from local farmers whose farms were not in the FP and typically raised them for approxi mately 18 months. He had four to six cows on the property at a time, and the cows routinely roamed and grazed in areas that flooded annually. They ate a mixture of FP grass, grain not grown in the FP, and hay from a nearby field that was near the FP but did not flood. The cattle were slaughtered at a local commercial abattoir. The meat always passed federal inspection (based on visual inspection, no chemical testing), but the livers never passed (for unknown reasons), and no organ meats were consumed. The meat was never sold but was distributed to friends and family members. He stopped raising beef sometime in 1996. At the time of our follow-up investigation (2008), no meat samples were available for chemical analyses. Beginning in the early 1980s, he started a vegetable garden in an area of the TR FP that regularly flooded. Vegetables included asparagus, tomatoes, cucumbers, green beans, corn, radishes, potatoes, beets, green peppers, onions, Swiss chard, watermelon, and pumpkins. Many vegetables were canned and consumed year-round. The vegetables were shared with the same friends and family members. Use of the vegetable garden was largely discontinued in about 1997, except for occasional tomato plants and asparagus. No one else among the 946 subjects in the parent study (who were a representative sample of the adult population in the regions studied) reported such regular and prolonged consumption of beef from the TR FP.

UMDES study participants who lived along the TR had one near-river soil, and vegetation sample analyzed for PCDDs, PCDFs and PCBs. Because the cattle grazed over a relatively wide area along the river FP, averaging of soil and vegetation measurements obtained from properties in the vicinity of the index case (including the index case) would likely provide a better index of exposure of cattle to FP contamination. Mean 2,3,4,7,8-pentaCDF concentrations in soil and vegetation based on six near-river samples obtained from the vicinity of the index case’s property were 1,250 pg/g dry weight for soil (range, 14.4–3,790 pg/g dry weight) and 122 pg/g dry weight for vegetation (range, 4.66–315 pg/g dry weight). The corresponding concentration of 2,3,4,7,8-pentaCDF in beef calculated according to Equations 1 and 2 amounts to 1,780 pg/g lipid in beef lipid (range, 30–6,820 pg/g lipid) or 180 pg/g wet weight in beef meat (range, 3–680 pg/g wet weight). The beef lipid mean value is more than three orders of magnitude greater than the maximum concentration of 1.1 pg/g lipid observed for commercial beef in the United States. This finding indicated that the cows raised by the index case were likely heavily contaminated (Ferrario et al. 1996).

**Figure 2:**
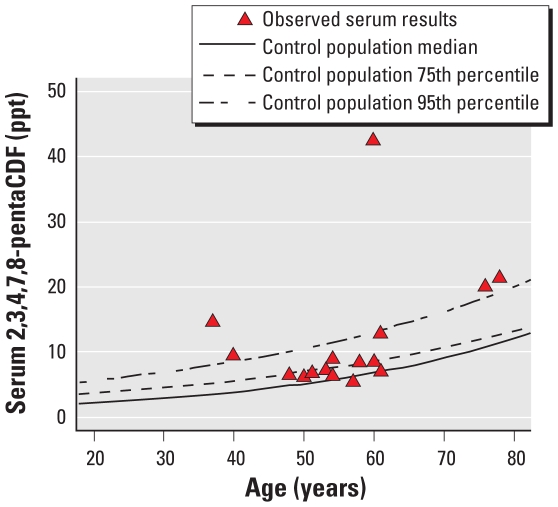
Serum 2,3,4,7,8-pentaCDF results by age with quantile curves based on the UMDES control population.

The index case had the greatest disparity between his serum concentration of 2,3,4,7,8-pentaCDF and the predicted value for someone of his age and BMI (42.5 ppt, or 4.29 studentized residuals above the predicted value based on the UMDES referent population). One other subject (case 2) had a serum concentration of 2,3,4,7,8-pentaCDF that was more than three studentized residuals above the predicted value (14.7 ppt, or 3.48 studentized residuals above the mean for those of comparable age and BMI); all remaining subjects’ serum concentrations of 2,3,4,7,8-pentaCDF were < 2.5 studentized residuals above their respective predicted values [Table 1; for complete serum results for all 16 subjects, see Supplemental Material, Table 1 (doi:10.1289/ehp.0901723)]. The mean serum concentration of 2,3,4,7,8-pentaCDF was 12.0 ppt among the 16 subjects (9.94 excluding the index case). For comparison, the overall mean, median, 95th percentile, and maximum for serum 2,3,4,7,8-pentaCDF in the UMDES control population were 6.0 ppt, 5.4 ppt, 13.0 ppt, and 26.2 ppt, respectively (not adjusted for age or BMI). Figure 2 displays the serum 2,3,4,7,8-pentaCDF levels among the 16 cases, along with quantile curves based on the control population (median, 75th percentile, and 95th percentile curves). None of the 16 subjects had an occupational history that might have provided opportunity for exposure to 2,3,4,7,8-pentaCDF, and none of them ever lived near a source of aerosol emission of furans or other dioxins. Previous work has shown that merely living on soil with 2,3,4,7,8-pentaCDF, (and house dust with contamination) makes no contribution to serum (Garabrant et al. 2009b).

Table 1 includes an estimate of the percentage contribution to each subject’s total serum TEQ from “extra” 2,3,4,7,8-pentaCDF, that is, the amount of 2,3,4,7,8-pentaCDF in each individual’s serum above his or her predicted value based on the referent population after adjusting for age, age squared, and BMI. The percentage contribution of extra 2,3,4,7,8-pentaCDF to the TEQ ranges from −1.7% to 20.4%, with a mean and median contribution of 4.9% and 2.6%, respectively, among the 16 cases.

Subjects were asked about the average number of beef and vegetable meals consumed per week or per month during the time period in which they ate the beef or vegetables from the index case. The estimated number of beef meals ranged from zero to 2,248, with a mean and median of 1,109 and 1,056 beef meals, respectively [see Supplemental Material, Table 2 (doi:10.1289/ehp.0901723)]. The estimated number of meals with vegetables from the garden of the index case ranged from 44 to 1,350, with a mean and median of 570 and 506 meals, respectively.

Because consumption of potentially contaminated beef stopped in 1996 (and most consumption of vegetables stopped in about 1997), but serum measurements were not performed until 2008 (2005 for the index case, 2004 for case 5), it is possible that the present results underestimate past serum levels of 2,3,4,7,8-pentaCDF. To illustrate the potential magnitude of such attenuation with time, the “excess” serum level of 2,3,4,7,8-pentaCDF in 1996 was estimated for the index case. Considering the BMI, the age, and the smoking status of the index case yields an estimated average elimination half-life of τ_1/2_ = 4.4 years. With this half-life estimate, the steady-state serum 2,3,4,7,8-pentaCDF concentration in 1996 was estimated to be 4.1 times higher than when it was measured in 2005. His serum concentration increase of 2,3,4,7,8-pentaCDF in 1996 due to prior consumption of contaminated beef and/or vegetables was therefore estimated to be ΔC_serum_ = 4.1 × (2,3,4,7,8-pentaCDF in 2008 – predicted based on UMDES controls) = 4.1 × (42.5 – 6.8) = 146 pg/g lipid, corresponding to a potential increase of 146 × 0.3 (the TEF for 2,3,4,7,8-pentaCDF) = 44 pg/g lipid in total serum TEQ.

## Discussion

Most cases included in this follow-up investigation had higher serum concentrations of 2,3,4,7,8-pentaCDF than predicted from the control population (adjusted for age, age squared, and BMI), but with two exceptions, all were less than 2.5 studentized residuals above their mean values. The estimated extra or excess contribution of 2,3,4,7,8-pentaCDF to the TEQ averaged 4.9% and was less than 21% in all cases. The results suggest that pro-longed regular consumption of beef and/or vegetables raised in the TR FP, a region documented to have widespread and high levels of dioxin contamination of FP sediments, can be a completed pathway of human exposure. In the present case, because of attenuation linked to the elapsed time between beef consumption and serum measurements, the estimated contribution of such past exposure to current TEQ above background appears to be modest (< 21% in all cases), adding, on average, less than 5% to the serum TEQ. However, estimated steady-state serum 2,3,4,7,8-pentaCDF concentrations in 1996 would have been as much as 4.1 times higher than in 2005 and would have added 44 ppt to the total serum TEQ of the index case.

A key element of this study was the remote dietary recall of the participants who consumed the beef and vegetables raised on the property of the index case. Subjects were asked to recall the frequency of meals consumed per week or per month with the beef and vegetables from 12 to 22 years before the interviews. Although subjects’ recall of whether they ever ate the beef and the vegetables, and the approximate time frame of such consumption, may be reasonably reliable, recall of frequency of remote consumption of specific foods is known to be less reliable and may explain some of the observed variation in serum levels of 2,3,4,7,8-pentaCDF (Wu et al. 1988). This uncertainty appears to be unavoidable in the present circumstance.

Cattle that forage on dioxin-contaminated soil can take up and bioaccumulate such contamination, which can then be passed on to the human consumers (Chang et al. 1989). For most vegetables, the transfer and bio-accumulation of dioxins in contaminated soil to the leaves or fruit are generally low (Hülster and Marschner 1993; Hülster et al. 1994; Muller et al. 1994). However, for some vegetables in the family Cucurbitaceae, such as pumpkin and zucchini, uptake from soil can be considerable (Hülster et al. 1994). No measurements of dioxins in meat or vegetables were performed as part of this investigation, and information about the frequency of consuming specific vegetables from the garden of the index case was not obtained. Based on the knowledge that most vegetables do not become significantly contaminated from soil, we assumed that consuming the beef raised on the property of the index case was the dominant pathway of exposure to dioxins in FP soil for the subjects of this study. Other investigators have also suggested that consuming animal products is a more important pathway of exposure than consuming vegetables raised on contaminated soil (Ewers et al. 1997).

Food is the dominant route of exposure to dioxins for most people. A number of studies have documented levels of PCDDs, PCDFs, and PCBs in fish destined for human consumption and higher serum levels in people from consuming fish raised in contaminated regions (Hites et al. 2004; Lee et al. 2006). In other cases, animal feed was contaminated from one of a variety of sources (e.g., ball clay added as an anticaking agent to soy meal in feed; citrus pulp used in cattle feed; oil contaminated with PCDDs, PCDFs, and PCBs added to recycled fat that was added to animal feed), which resulted in contamining chicken, beef, and catfish and subsequently contamining humans (Huwe 2002). Rogan (1982) reported that cooking oil that was contaminated with PCBs and PCDFs resulted in outbreaks of illness in Taiwan and Japan. There have also been a few investigations of human exposure to dioxins from consumption of farm animals raised on contaminated soil (Ewers et al. 1996, 1997).

## Relevance to Public Health Practice

The subjects in the present study reported regularly consuming beef and vegetables raised in a region heavily contaminated with dioxins. Their dietary practices were unusual if not unique: No one else among the 946 subjects in the parent study (a representative sample of the adult population in the regions studied) reported such regular and prolonged consumption of beef and vegetables raised in the TR FP. The dietary practices of the index case (and friends and family members who also ate the beef and vegetables) appear to be a “worst-case scenario” in terms of completing a pathway of exposure to dioxin contamination in TR FP soil and achieving the highest serum level of 2,3,4,7,8-pentaCDF (after adjusting for age, age squared, and BMI) in the entire study. Although this is a case study and other exposure sources and path-ways cannot be excluded, the estimated impact of consumption of these homegrown foods on current serum TEQ was modest (i.e., contributed < 5% to the serum TEQ on average and < 21% to the serum TEQ in all cases); it likely was much greater in the past. The results of this study indicate that animals and crops should not be raised for human consumption in areas contaminated with dioxins.

## Figures and Tables

**Figure 1:**
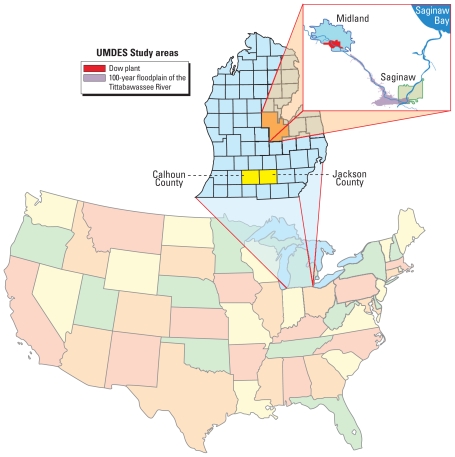
Map of UMDES areas.

**Table 1:** TEQ and 2,3,4,7,8-pentaCDF concentrations in serum among people who consumed beef and vegetables raised by the index case.

Case	Age at blood draw (years)	WHO-TEQ (ppt)	WHO-TEQ studentized residuals^a^	2,3,4,7,8-PentaCDF (ppt)	PentaCDF studentized residuals^a^	Predicted pentaCDF^b^ (ppt)	Percent contribution of excess 2,3,4,7,8-pentaCDF to TEQ
1^c^	≥ 60	52.4	2.22	42.5	4.29	6.8	20.4
2	30–44	17.6	1.21	14.7	3.48	3.5	19.3
3	30–44	18.1	1.02	9.5	2.25	3.8	9.4
4	≥ 60	44.0	0.32	21.4	1.79	9.9	7.9
5	≥ 60	69.4	1.45	20.1	1.59	10.2	4.3
6	≥ 60	33.1	0.81	12.9	1.47	7.2	5.1
7	45–59	24.1	0.53	8.84	1.02	6.0	3.5
8	45–59	24.3	0.21	8.42	0.63	6.7	2.1
9	45–59	13.7	−0.41	6.34	0.69	4.9	3.1
10	45–59	31.0	1.02	7.11	0.41	6.4	0.7
11	45–59	19.6	0.02	6.72	0.46	5.9	1.3
12	≥ 60	22.5	−0.29	8.45	0.45	7.3	1.5
13	45–59	14.8	−0.46	6.03	0.38	5.4	1.4
14	45–59	20.4	0.10	6.29	0.21	6.0	0.5
15	≥ 60	19.3	−0.72	6.94	−0.05	7.3	−0.6
16	45–59	19.2	−0.28	5.34	−0.37	6.4	−1.7

WHO (World Health Organization) TEQs were calculated as described by Van den Berg et al. (2006).

a Distance from the lognormal mean of the referent population after adjusting for age, age squared, and BMI.

b Predicted mean 2,3,4,7,8-pentaCDF (in parts per trillion) for persons with the same age and BMI of each subject based on the referent population.

c Index case.

## References

[b1-ehp-118-1313] ATSDR (Agency for Toxic Substances and Disease Registry) (1994). Toxicological Profile for Chlorodibenzofurans (CDFs).

[b2-ehp-118-1313] ATSDR (Agency for Toxic Substances and Disease Registry) (1998). Toxicological Profile for Chlorinated Dibenzo-p-dioxins (CDDs).

[b3-ehp-118-1313] ATSDR (Agency for Toxic Substances and Disease Registry) (2000). Toxicological Profile for Polychlorinated Biphenyls (PCBs).

[b4-ehp-118-1313] Chang R, Hayward D, Goldman L, Harnly M, Flattery J, Stephens R (1989). Foraging farm animals as biomonitors for dioxin contamination. Chemosphere.

[b5-ehp-118-1313] Demond A, Adriaens P, Towey T, Chang SC, Hong B, Chen Q (2008). Statistical comparison of residential soil concentrations of PCDDs, PCDFs and PCBs from two communities in Michigan. Environ Sci Technol.

[b6-ehp-118-1313] Ewers U, Wittsiepe J, Hens-Bischoff G, Balzer W, Alger B, Urban U (1997). Human biomonitoring—studies of arsenic, lead and PCDD/F in inhabitants of a contaminated residential area. Gesundheitswesen.

[b7-ehp-118-1313] Ewers U, Wittsiepe J, Schrey P, Gatzert U, Hinz S, Csicsaky M (1996). Blood PCDD/F levels in blood of residents of a former cable incineration facility. Gesundheitswesen.

[b8-ehp-118-1313] Feil VJ, Huwe JK, Zaylskie RG, Davison KL, Anderson VL, Marchello M (2000). Chlorinated dibenzo-*p*-dioxin and dibenzofuran concentrations in beef animals from a feeding study. J Agric Food Chem.

[b9-ehp-118-1313] Ferrario JB, Byrne CJ (2000). 2,3,7,8-Dibenzo-*p*-dioxins in mined clay products from the United States: evidence for possible natural origin. Environ Sci Technol.

[b10-ehp-118-1313] Ferrario J, Byrne C, McDaniel D, Dupuy A, Harless R (1996). Determination of 2,3,7,8 chlorine (Cl)-substituted dibenzo-*p*dioxins and furans at the part per trillion level in United States beef fat using high resolution gas chromatography/high resolution mass spectrometry. Anal Chem.

[b11-ehp-118-1313] Franzblau A, Hedgeman E, Chen Q, Lee SY, Adriaens P, Demond A (2008). Case report: human exposure to dioxins from clay. Environ Health Perspect.

[b12-ehp-118-1313] Garabrant DH, Franzblau A, Lepkowski J, Gillespie BW, Adriaens P, Demond A (2009a). The University of Michigan Dioxin Exposure Study: methods for an environmental exposure study of polychlorinated dioxins, furans and biphenyls. Environ Health Perspect.

[b13-ehp-118-1313] Garabrant DH, Franzblau A, Lepkowski J, Gillespie BW, Adriaens P, Demond A (2009b). The University of Michigan Dioxin Exposure Study: predictors of human serum dioxin concentrations in Midland and Saginaw, Michigan. Environ Health Perspect.

[b14-ehp-118-1313] Hilscherova K, Kannan K, Nakata H, Hanari N, Yamashita N, Bradley PW (2003). Polychlorinated dibenzo-*p*-dioxin and dibenzofuran concentration profiles in sediments and floodplain soils of the Tittabawassee River, Michigan. Environ Sci Technol.

[b15-ehp-118-1313] Hites RA, Foran JA, Carpenter DO, Hamilton MC, Knuth BA, Schwager SJ (2004). Global assessment of organic contaminants in farmed salmon. Science.

[b16-ehp-118-1313] Hülster A, Marschner H (1993). Transfer of PCDD/PCDF from contaminated soils to food and fodder crop plants. Chemosphere.

[b17-ehp-118-1313] Hülster A, Muller JF, Marschner H (1994). Soil-plant transfer of polychlorinated dibenzo-*p*-dioxins and dibenzofurans to vegetables of the cucumber family (Cucurbitaceae). Environ Sci Technol.

[b18-ehp-118-1313] Huwe JK (2002). Dioxins in food: a modern agriculture perspective. J Agric Food Chem.

[b19-ehp-118-1313] IAEA (1994). Handbook of Parameter Values for the Prediction of Radionuclide Transfer in Temperate Environments.

[b20-ehp-118-1313] Lee CC, Lin WT, Liao PC, Su HJ, Chen HL (2006). High average daily intake of PCDD/Fs and serum levels in residents living near a deserted factory producing pentachlorophenol (PCP) in Taiwan: influence of contaminated fish consumption. Environ Pollut.

[b21-ehp-118-1313] Milbrath MO, Wenger Y, Chang CW, Emond C, Garabrant D, Gillespie BW (2009). Apparent half-lives of dioxins, furans, and PCBs as a function of age, body fat, smoking status, and breastfeeding. Environ Health Perspect.

[b22-ehp-118-1313] Müller JF, Hülster A, Päpke O, Ball M, Marschner H (1994). Transfer of PCDD/PCDF from contaminated soils into carrots, lettuce, and peas. Chemosphere.

[b23-ehp-118-1313] Rogan WJ (1982). PCBs and cola-colored babies: Japan, 1968, and Taiwan, 1979. Teratology.

[b24-ehp-118-1313] U.S. EPA (Environmental Protection Agency) (1994). Method 8290. Polychlorinated Dibenzodioxins (PCDDs) and Polychlorinated Dibenzofurans (PCDFs) by High-Resolution Gas Chromatography/High-Resolution Mass Spectrometry (HRGC/HRMS).

[b25-ehp-118-1313] U.S. EPA (Environmental Protection Agency) (1999). Method 1668, Revision A: Chlorinated Biphenyl Congeners in Water, Soil, Sediment, and Tissue by HRGC/HRMS.

[b26-ehp-118-1313] Van den Berg M, Birnbaum LS, Denison M, De Vito M, Farland W, Feeley M (2006). The 2005 World Health Organization reevaluation of human and mammalian toxic equivalency factors for dioxins and dioxin-like compounds. Toxicol Sci.

[b27-ehp-118-1313] Wu ML, Whittemore AS, Jung DL (1988). Errors in reported dietary intakes. II. Long-term recall. Am J Epidemiol.

